# Prevalence and associated factors of antenatal depression of women attending antenatal clinics in two tertiary care maternity hospitals in Sri Lanka

**DOI:** 10.1192/bjo.2021.776

**Published:** 2021-06-18

**Authors:** Chathurie Suraweera, Iresha Perera, L.L. Amila Isuru, Janith Galhenage

**Affiliations:** 1 Professorial Psychiatry Unit, National Hospital of Sri Lanka; 2 Faculty of Medicine and Allied Sciences, Rajarata University of Sri Lanka

## Abstract

**Aims:**

To determine the prevalence of depression among antenatal mothers in two tertiary care maternity hospitals in Colombo and associated factors of antenatal depression.

**Method:**

A descriptive cross-sectional study was conducted in antenatal clinics in two tertiary care maternity hospitals in Sri Lanka. Every second woman attending the clinic was recruited using systematic sampling until the calculated sample size was obtained. A structured questionnaire and Edinburgh Postnatal Depression Scale (EPDS) were used for data collection. Data were analysed using SPSS.

**Result:**

A total of 536 pregnant women were participated in the study. Around one third (180, 33.6%) of pregnant women had depression according to the EPDS score (Mean = 7.66, SD = 5.17). The mean age was 29.65(SD = 9.30) years and among them 387(74.3%) had at least secondary education. The majority lived with parents or in-laws (329, 61.4%) in addition to nuclear family members and 266(49.6%) were in their third trimester. Most women were in first pregnancy (149, 39.5%) and 11 (2.1%) had a history of psychiatric illness. Sixty (11.2%) women and 156(29.1%) of partners used psychoactive substances. Verbal abuse and physical abuse were reported by 5(0.9%) and 3(0.6%) respectively. Sixty one (11.4%) women have reported inadequate family support, and 226 (42.2%) had only support of the partner. Among them 346 (64.6%) reported excellent support from partner. One third (186, 34.7%) of pregnancies were unplanned, 328(61.2%) women reported a very good relationship with their partner. Complications were experienced by 123(22.9%) of women during this pregnancy and commonest was gestational diabetes. The presence of depression was significantly associated with living with extended family (p = 0.033) and in-laws(p = 0.014). Multi parity (>2 children) (p = 0.008), partner's substance use (p = 0.002), inadequate family support (p = 0.024), inadequate partner's support (p = 0.003), unsatisfactory relationship with partner (p = 0.000) and unplanned pregnancies (p = 0.001) were also associated with depression. Logistic regression analysis indicated a significant association between depression with partner's substance use, unavailability of family support and poor relationship with the partner.

**Conclusion:**

Around one-third of mothers were having antenatal depression. Several spouse related factors and unsatisfactory family support were associated with depression among antenatal mothers.

